# Serum Interleukin-6 and -8 as Predictors of Response to Vedolizumab in Inflammatory Bowel Diseases

**DOI:** 10.3390/jcm9051323

**Published:** 2020-05-02

**Authors:** Lorenzo Bertani, Gian Paolo Caviglia, Luca Antonioli, Rinaldo Pellicano, Sharmila Fagoonee, Marco Astegiano, Giorgio Maria Saracco, Elisabetta Bugianesi, Corrado Blandizzi, Francesco Costa, Davide Giuseppe Ribaldone

**Affiliations:** 1 Department of Translational Research and New Technologies in Medicine and Surgery, University of Pisa, 56100 Pisa, Italy; lorenzobertani@gmail.com; 2 Department of Medical Sciences, University of Turin, 10126 Turin, Italy; giorgiomaria.saracco@unito.it (G.M.S.); elisabetta.bugianesi@unito.it (E.B.); 3 Department of Clinical and Experimental Medicine, University of Pisa, 56126 Pisa, Italy; lucaant@gmail.com (L.A.); c.blandizzi@gmail.com (C.B.); 4 Unit of Gastroenterology, Molinette Hospital, 10126 Turin, Italy; rinaldo_pellican@hotmail.com (R.P.); marcoastegiano58@gmail.com (M.A.); 5 Institute of Biostructure and Bioimaging, CNR c/o Molecular Biotechnology Centre, 10126 Turin, Italy; sharmila.fagoonee@unito.it; 6 Department of General Surgery and Gastroenterology, IBD Unit, Pisa University Hospital, 56100 Pisa, Italy; fcosta@med.unipi.it

**Keywords:** clinical, Crohn’s disease, cytokines, serum, ulcerative colitis

## Abstract

Vedolizumab, a monoclonal antibody directed against integrin α4β7, is an effective treatment for inflammatory bowel diseases. However, a significant number of patients do not achieve steroid-free clinical remission in the first year of treatment. An early identification of these patients is one of the most important challenges for clinicians and offers the possibility of therapeutic optimization in order to personalize biological therapy. The aim of our study was to test the prediction ability of interleukin (IL)-6 and -8 of clinical response after 12 months of therapy with vedolizumab (T2). We performed a prospective, multicentre study in patients affected by inflammatory bowel disease by analysing cytokines level before starting vedolizumab (T0) and after 10 weeks of therapy (T1). In the overall cohort (*n* = 54), IL-8 decrease > 2.6 pg/mL in the first 10 weeks of therapy was able to predict clinical response (area under the curve (AUC) = 0.70, sensitivity = 66%, specificity = 75%, *p* = 0.010), negative C-reactive protein (CRP) (AUC = 0.71, sensitivity = 64%, specificity = 80%, *p* = 0.009) and calprotectin < 250 mg/kg (AUC = 0.69, sensitivity = 64%, specificity = 78%, *p* = 0.030) after 44 weeks of therapy. In patients with ulcerative colitis (*n* = 40), baseline IL-8 values > 8.6 pg/mL and a decrease of IL-6 values > 0.4 pg/mL from T0 to T1 were significant and independent predictors of clinical response after 12 months of vedolizumab therapy (odds ratio (OR) = 6.96, 95% CI 1.27–38.22, *p* = 0.026 and OR = 7.29, 95% CI 1.42–37.50, *p* = 0.017, respectively). In patients with Crohn’s disease (*n* = 14), baseline IL-8 values > 8.6 pg/mL and baseline IL-6 values > 1.6 pg/mL allowed the identification of patients achieving negative CRP at T2 (AUC = 0.75, sensitivity = 74%, specificity = 76%, *p* < 0.001) and patients with faecal calprotectin values < 250 mg/kg at T2 (AUC = 0.71, sensitivity = 78%, specificity = 63%, *p* = 0.004). In conclusion, our study highlights a potential clinical role of serum cytokine levels for the prediction of clinical and biochemical steroid-free response in patients treated with vedolizumab.

## 1. Introduction

Inflammatory bowel diseases (IBD) are chronic gastrointestinal disorders consisting of two main entities, ulcerative colitis (UC) and Crohn’s disease (CD), both characterized by an immune-mediated pathogenesis and a clinical relapsing course [1,2].

In the last two decades, a more comprehensive understanding of the cytokine pathways involved in the pathogenesis of IBD allowed the development of new treatment strategies that led to reduced use of corticosteroids [3]. Vedolizumab (VDZ) is an important therapeutic option for IBD patients [4], due to a different mechanism of action, as compared to nonbiological therapeutic approaches or anti-tumour necrosis factor (TNF) agents [5]. This monoclonal antibody, which binds α4β7-integrin expressed in a subset of T-lymphocytes, prevents the adherence and diapedesis of the latter through the mucosal vascular addressin cell adhesion molecule (MAdCAM)-1, expressed only in the gut endothelium [6]. Despite a satisfactory clinical development programme, real-life studies reveal that only 40% of patients treated with VDZ achieve clinical remission [7].

Therefore, it is crucial to identify biomarkers able to predict and monitor therapeutic success in order to tailor individualized treatment strategies. The most used biomarker in IBD practice is faecal calprotectin, but it reflects only a non-specific anti-inflammatory response [8,9]. For this reason, an analysis of cytokine levels in patients with IBD could be useful to predict the pharmacological response to treatment with biological drugs, such as VDZ. In this perspective, the most relevant cytokines involved in the pathophysiology of IBD [10,11,12] and studied as possible biomarkers of therapeutic response [13,14,15,16] are interleukin (IL)-6 and IL-8. Indeed, in a previous monocentre study involving patients with UC and CD treated with different types of biologics drugs, we observed that IL-6 reduction from baseline to 10 weeks of treatment was able to broadly predict clinical response at 12 months of therapy [17].Here, we aimed at investigating the prediction ability of IL-6 and -8 of clinical response after 10 weeks and after 12 months of therapy with VDZ.

## 2. Materials and Methods

We performed a prospective, multicentre study at the Gastroenterology Unit of “Città della Salute e della Scienza di Torino”, Italy and at IBD Unit, Pisa University Hospital, Pisa, Italy. From January 2018 to January 2019 we recruited consecutive patients: (1) affected by IBD with indications to treatment with VDZ; (2) older than or equal to 18 years; (3) who agreed to sign the informed consent to participate in the study. We treated patients with moderate-to-severe disease activity or steroid-dependent disease with previous failure or intolerance to thiopurines with VDZ [18,19]. Exclusion criteria were: (1) refusal to participate to the study.

Clinical history, data on physical examination, recent biochemical examinations and signed informed consent for the purpose of enrolment in the study, were collected. All patients were treated with a total dose of 300 mg by infusion at 0–2–6 weeks interval as induction, and every 8-weeks thereafter during the year. In particular, none of the patients affected by CD was treated with the infusion at week 10 and none was treated with a 4- or 6-weeks maintenance regimen. Before starting VDZ therapy, venous blood was collected. The blood samples were associated with a numerical identification code and stored frozen at −80 °C. Two blood samples were collected from each IBD patient, the first before the start of VDZ therapy (T0) and the second after 10 weeks of treatment (T1).

IL-6 and IL-8 were measured in serum samples by Bio-Plex^®^ Multiplex Immunoassay (Bio-Rad Laboratories, Pleasanton, CA, USA) on Luminex^®^ 200 system (Luminex Corporation, Austin, TX, USA) [20]. Individual standard curves were generated for each cytokine; results were given in pg/mL.

### 2.1. Description of the Cohort

The cohort included 54 patients. The epidemiological characteristics, clinical, biochemical and endoscopic activity, medications of the recruited patients are reported in Table 1.

All patients with CD were previously treated with anti-TNF therapy (adalimumab), except one due to contraindication to these drugs; 24 patients with UC were previously treated with anti-TNF therapy (infliximab), 16 were naive to biological therapies. No patients were previously treated with ustekinumab or tofacitinib.

The primary aim was to evaluate the prediction ability of the trends observed in IL-6 and IL-8 levels between T0 and T1 of clinical response at twelve months of therapy. The secondary goals were to evaluate: (1) the prediction ability of IL-6 and IL-8 at T0 of clinical response at T2; (2) the prediction ability of baseline levels and trends of IL-6 and IL-8, of negative C-reactive protein (CRP) and calprotectin values < 250 mg/kg at twelve months of therapy; (3) sub-analyses for CD and UC.

Clinical response to VDZ therapy was defined as a decrease in the Harvey-Bradshaw index (HBI) score greater than or equal to 3 (or HBI ≤ 4 at month twelve) or in the partial Mayo (pMAYO) score greater than or equal to 2 (or pMAYO ≤ 1 at month twelve), in absence of corticosteroid therapy and with ongoing VDZ therapy, in agreement with literature [21].

The study followed the principles of the Declaration of Helsinki and was approved by the local ethical committee (Comitato Etico Interaziendale A.O.U. Città della Salute e della Scienza di Torino—A.O. Ordine Mauriziano—A.S.L. Città di Torino) (approval code 0056924).

### 2.2. Statistical Analysis

Continuous variables were reported as mean (range or 95% confidence interval (CI)), geometric mean or as median depending on data distribution. The normality of the data was evaluated by D’Agostino-Pearson test. We performed an intention-to-treat analysis and included all the patient that started VDZ in the final analysis. The comparison of continuous variables between independent groups was performed by independent samples *t*-test. The comparison of continuous paired measurements was carried out by *t*-student test for paired measurements or by Wilcoxon test, depending on data distribution. The comparison of paired, dichotomous qualitative variables was carried out by McNemar test. Receiver operating characteristic (ROC) curve analysis for used to test the ability of IL-6 and IL-8 to discriminate between patients who achieved the outcomes from those who did not. Diagnostic accuracy has been reported as area under the curve (AUC) value. Logistic regression was performed to derive the odds ratio (OR), with its 95% confidence interval, as a measure of the strength of association between two variables.

Since no previous studies have analysed the ability of IL-6 and IL-8 to predict clinical response to VDZ in patients with CD and UC, a priory calculation of the power of the study has not been possible. We chose to include twice the number of patients recruited in the most similar study (27 patients; cytokines’ prediction ability of mucosal healing in UC [16]). 

A *p*-value of less than 0.05 was considered significant. Statistical analysis was performed with MedCalc Statistical Software version 18.9.1 (MedCalc Software bvba, Ostend, Belgium; http://www.medcalc.org; 2018).

## 3. Results

IL-6 and IL-8 baseline values are reported in Table 2.

The trends of the parameters at 3 and 12 months in the overall cohort and according to type of disease are reported in Table 3.

**Figure 1:**
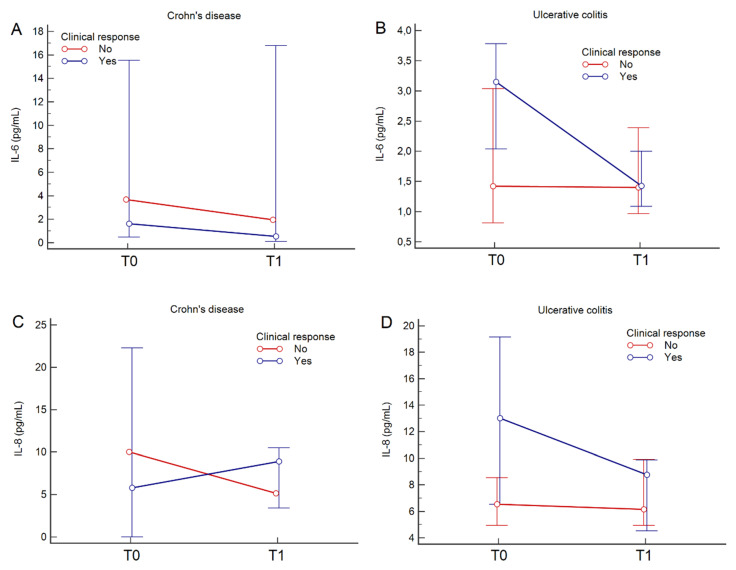
IL-6 and IL-8 variation from T0 to T1 in patients with CD (**A**,**C**) and UC (**B**,**D**) according to clinical response to treatment.

IL-6 values from T0 to T1 did not change in CD (median value from 1.6 to 2.0 pg/mL, *p* = 0.414) and decreased in UC (median value from 2.5 to 1.4 pg/mL, *p* = 0.009); IL-8 values did not change in CD (median values from 5.8 to 8.9 pg/mL, *p* = 0.970) and decreased in UC (median values from 8.3 to 8.0 pg/mL, *p* = 0.032). IL-6 and IL-8 variation from T0 to T1 classified according to treatment response and to type of disease (CD or UC) are depicted in Figure 1.

Seven patients stopped VDZ during the year due to drug failure. These patients, as stated in the Material and Methods, were considered as failure of primary outcome (clinical response to VDZ therapy was defined as a decrease in the HBI score greater than or equal to 3 (or HBI ≤ 4) or in the pMAYO score greater than or equal to 2 (or pMAYO ≤ 1), in absence of corticosteroid therapy and with ongoing VDZ therapy). At T2 33/54 (61.1%) patients achieved clinical response. In the overall cohort of patients with IBD, we observed that IL-8 reduction > 2.6 pg/mL from T0 to T1 was able to discriminate between patients who responded to therapy at T2 from those who did not (AUC = 0.70, sensitivity = 66%, specificity = 75%, *p* = 0.010). Baseline IL-8 values > 8.6 pg/mL and IL-8 reduction > 2.6 pg/mL from T0 to T1 were able to identify patients achieving CRP negativization at T2 (AUC = 0.70, sensitivity = 74%, specificity = 76%, *p* = 0.021 and AUC = 0.71, sensitivity = 64%, specificity = 80%, *p* = 0.009, respectively). Baseline IL-6 values > 1.6 pg/mL and IL-8 reduction > 2.6 pg/mL from T0 to T1 were able to identify patients achieving faecal calprotectin values < 250 mg/kg at T2 (AUC = 0.70, sensitivity = 78%, specificity = 74%, *p* = 0.020 and AUC = 0.69, sensitivity = 64%, specificity = 78%, *p* = 0.030, respectively).

In patients with CD, we observed that baseline IL-8 values > 8.6 pg/mL allowed the identification of patients achieving negative CRP at T2 (AUC = 0.75, sensitivity = 74%, specificity = 76%, *p* < 0.001) while baseline IL-6 values > 1.6 pg/mL identified patients with faecal calprotectin values < 250 mg/kg at T2 (AUC = 0.71, sensitivity = 78%, specificity = 63%, *p* = 0.004).

**Figure 2:**
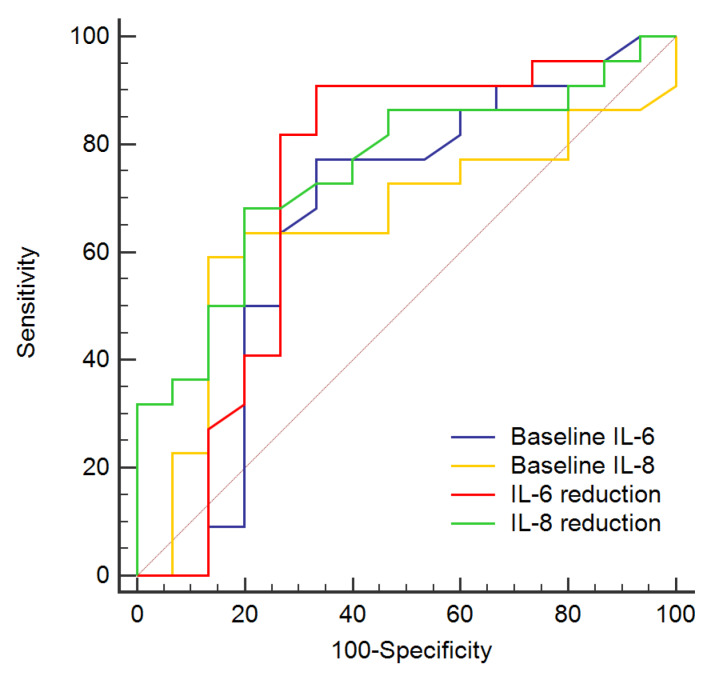
ROC curve of baseline IL-6 values, baseline IL-8 values, IL-6 reduction from T0 to T1 and IL-8 reduction from T0 to T1 for the identification of patients with UC that achieved a clinical response to therapy at T2.

In patients with UC, baseline IL-6 values > 1.6 pg/mL allowed the identification of patients achieving a clinical response at 12 months of therapy (AUC = 0.70, sensitivity = 79%, specificity = 60%, *p* = 0.012) and faecal calprotectin values < 250 mg/kg at T2 (AUC = 0.71, sensitivity = 79%, specificity = 60%, *p* = 0.006). Baseline IL-8 values > 8.6 pg/mL identified patients who achieved a clinical response at 12 months of treatment (AUC = 0.70, sensitivity = 58%, specificity = 80%, *p* = 0.010), negative CRP at T2 (AUC = 0.73, sensitivity = 68%, specificity = 78%, *p* = 0.002) and faecal calprotectin values < 250 mg/kg at T2 (AUC = 0.70, sensitivity = 65%, specificity = 75%, *p* = 0.011). IL-6 reduction > 0.4 pg/mL from T0 to T1 led to the identification of patients who achieved a clinical response at 12 months of treatment (AUC = 0.73, sensitivity = 73%, specificity = 73%, *p* = 0.003), negative CRP at T2 (AUC = 0.72, sensitivity = 74%, specificity = 71%, *p* = 0.004) and faecal calprotectin values < 250 mg/kg at T2 (AUC = 0.78, sensitivity = 75%, specificity = 80%, *p* < 0.001). IL-8 reduction > 2.6 pg/mL from T0 to T1 identified patients achieving a clinical response at 12 months of treatment (AUC = 0.72, sensitivity = 64%, specificity = 80%, *p* = 0.004), negative CRP at T2 (AUC = 0.75, sensitivity = 68%, specificity = 82%, *p* < 0.001) and faecal calprotectin values < 250 mg/kg at T2 (AUC = 0.73, sensitivity = 65%, specificity = 80%, *p* = 0.003) (Figure 2).

To confirm that the event was specific to VDZ treatment, we performed a sub-analysis of cases with start or dose escalation of systemic corticosteroid within 2-weeks before or after the start of VDZ therapy. We excluded one patient from CD cohort and three patients from UC cohort (in total four patients from the whole IBD cohort). All the analyses confirmed that the effects observed were due to VDZ treatment and not caused by the steroid’s treatment.

By logistic regression analysis, we observed that baseline IL-6 values > 1.6 pg/mL (OR = 5.70, 95% CI 1.37–23.76, *p* = 0.017), baseline IL-8 values > 8.6 pg/mL (OR = 5.60, 95% CI 1.25–25.17, *p* = 0.025), IL-6 reduction > 0.4 pg/mL (OR = 7.33, 95% CI 1.67–32.21, *p* = 0.008) and IL-8 reduction > 2.6 pg/mL (OR = 7.00, 95% CI 1.51–32.48, *p* = 0.013) were significantly associated to clinical response at T2 in patients with UC, while neither CRP reduction (OR = 0.40, 95% CI 0.08–1.89, *p* = 0.248) nor faecal calprotectin reduction (OR = 4.52, 95% CI 0.85–24.11, *p* = 0.077) predicted clinical response. By multiple stepwise logistic regression analysis, only baseline IL-8 values > 8.6 pg/mL (OR = 6.96, 95% CI 1.27–38.22, *p* = 0.026) and IL-6 reduction > 0.4 pg/mL (OR = 7.29, 95% CI 1.42–37.50, *p* = 0.017) resulted significant and independent predictors of clinical response at T2. This result was further confirmed following adjustment for disease activity and extent (E3, *n* = 21, versus E1, *n* = 3 plus E2, *n* = 16) (Table 4).

## 4. Discussion

VDZ is effective for the treatment of IBD, as demonstrated by several real-life studies [22,23,24]. However, a considerable number of patients, ranging between 50 and 75%, do not achieve steroid-free clinical remission during the first year of treatment [22,23,24]. An early identification of potentially non-responding patients is one of the most important challenges for clinicians, which may lead to a possible therapeutic optimization in order to personalize biological therapy.

In recent years, several studies were conducted in order to identify reliable biomarkers of therapeutic effectiveness. At present, the only parameter that predicts a worst therapeutic outcome to VDZ therapy with reasonable confidence is the previous exposure to anti-TNF drugs [25,26]. Moreover, patients with severe clinical activity at baseline are less likely to respond to VDZ [26], as well as patients who achieve a clinical response at week 6 often achieve steroid-free clinical remission after one year [22].

Moving to laboratory biomarkers, a prospective real-life study showed that a decrease in faecal calprotectin at week 14 was associated with clinical remission at one year. However, data regarding its use as a prospective biomarker in VDZ-treated patients are conflicting, since a post-hoc analysis of GEMINI-I trial showed that faecal calprotectin levels after the induction of VDZ therapy are not able to predict endoscopic response [27]. Conversely, faecal calprotectin showed in several studies in IBD setting a reliable correlation with endoscopic activity [28,29,30].

CRP is the most used biomarker in IBD, but a recent review showed that it is not reliable in predicting therapeutic outcome to VDZ [31]. On the other hand, Buer et al. [32] displayed how higher levels of CRP at baseline were correlated with lower plasma levels of VDZ at week 14, and this finding is particularly important since there are several studies that showed how the measurement of drug levels could be used as marker of therapeutic outcome. Indeed, higher levels of VDZ are associated with clinical, biochemical and endoscopic remission [33,34,35], but also with treatment persistency [36]. In this perspective, Boden et al. [37] have proposed the use of VDZ trough levels, α4β7 baseline expression and its receptor as possible biomarkers of therapeutic response, while Battat et al. [38] showed a correlation between serum α4β7 integrin concentration and therapeutic outcome. Unfortunately, these analyses are not widely available, and their use in clinical practice is unlikely.

The evaluation of serum cytokine profiles could represent a reliable and non-invasive tool to predict the therapeutic efficacy of biological drugs [39]. Our study showed that serum cytokines IL-6 and IL-8 could have a role in predicting therapeutic outcome to VDZ. In particular, the most important finding of our study was the association between baseline IL-8 values and IL-6 reduction in the first three months of treatment with clinical response at twelve months in UC patients, but not in CD, even at multiple stepwise logistic regression analysis. Interestingly, the reduction of CRP or faecal calprotectin in the same timeframe was not able to predict clinical response. Notably, at multivariate regression analysis, baseline IL-8 values and IL-6 reduction remained the only parameters associated with clinical response, taking into account also the severity and extent of disease. The results of the present study are in partial agreement with a previous single-centre study involving patients with IBD treated with different types of biologic drugs [17]; in both studies, the reduction of IL-6 in course of therapy was associated to the clinical response at 12 months of treatment. Herein, focusing on patients undergoing VDZ, we found a significant predictive value also for baseline IL-8 serum levels.

A recent study [16] in a small population of UC patients treated with VDZ showed that the decrease in IL-6 and IL-8 over the first 6 weeks of treatment correlated with mucosal healing after twelve months of treatment. Consistently, in the present study we showed that a decrease > 0.4 pg/mL of IL-6 and > 2.6 pg/mL of IL-8 levels in the first three months predicted faecal calprotectin < 250 mg/kg at twelve months in patients affected by UC. Although endoscopic assessment is currently recognized as the best therapeutic outcome [21], faecal calprotectin < 250 mg/kg is commonly considered as marker of endoscopic remission, both in paediatric [29] and adult patients [28]. Of note, this threshold was chosen even in the famous CALM trial where treatment escalation was decided based on faecal calprotectin levels > 250 mg/kg [40]. Another important finding of the present study is the correlation between baseline IL-6 and clinical response at twelve months in UC patients; although a trend was highlighted in the previous study [16] statistical correlation was not achieved probably due to the smaller number of patients. Moreover, the present study showed that the decrease in IL-6 and IL-8 levels in the first three months of therapy was correlated to normal values of CRP at twelve months, which was not evaluated in the previous study [16]. Hence, our results provided evidence in a larger cohort of patients in confirming that the decrease of these cytokines was able to predict a complete biochemical response in VDZ-treated patients with UC, but not in CD.

Although additional evidences are necessary to draw definitive conclusions, the present study allowed to demonstrate the presence of a different behaviour of serum cytokines useful to predict therapeutic response in UC compared to CD. IL-8, mainly released by macrophages and epithelial cells, exerts its pro-inflammatory functions by promoting the chemiotactic attraction of neutrophils into the inflammatory site [41]. In UC, unrestricted neutrophil activation may cause significant tissue damage that further fuel chronic inflammation [42]. By contrast, in CD, defective neutrophils may not be able to limit invasion by microorganisms, hence leading consequently to an uncontrolled inflammatory reaction [43].

A study by Rodriguez-Perálvarez et al. [44] in UC showed that serum levels of this cytokine were correlated with disease activity in terms of clinic, endoscopic and histologic findings. Moreover, lower IL-8 levels have been found in non-ulcerated mucosa of UC patients, as compared to mucosal ulcerations [45]. The decrease of IL-8 more consistent in therapy responders than in non-responders to VDZ treatment showed in the present study could be explained by an increased expression of α4β7 integrin driven by this cytokine. Indeed, Boden et al. [37] found higher basal α4β7 expression on CD4 and CD8 T-cells, which are activated by IL-8, in responders as compared with non-responders to VDZ therapy. Furthermore, a recent study by Zeissig et al. [46] suggested that the therapeutic efficacy of VDZ could be related to an effect on cells of mucosal innate immunity, such as macrophages, which are the main producers of IL-8. Conversely, the role of IL-8 in predicting therapeutic response seems to be less important in CD [47].

Concerning CD patients, although baseline serum levels of IL-6 and IL-8 were not able to discriminate clinical response, baseline IL-8 values > 8.6 pg/mL were able to predict a negative CRP value at twelve months. On the other hand, baseline IL-6 values > 1.62 pg/mL predicted faecal calprotectin < 250 mg/kg at twelve months, Concerning CRP, this could be expected, according to several studies that showed how CRP is more helpful in CD than in UC [48,49], and the correlation between IL-8 and this biomarker has been demonstrated [50] Conversely, the correlation between IL-6 and faecal calprotectin could be surprisingly, since faecal calprotectin is more reliable in UC than in CD [8]. However, IL-6 is a pleiotropic cytokine produced by innate immune and supporting stromal cells and activates adaptive T and B effector cells [51]. Indeed, IL-6 levels were demonstrated to be significantly higher in CD patients, compared to those with UC [52]. In this perspective, the assessment of IL-6 levels was proposed in several studies in order to predict therapeutic outcome in CD. However, the results were conflicting: Billiet et al. [53] showed that IL-6 concentrations decreased significantly in responders compared to primary non-responders to infliximab therapy, whereas Yarur et al. [47] reported that the assessment of IL-6 and IL-8 levels is not reliable in predicting therapeutic effectiveness of anti-TNF drugs in CD.

Our study showed a preliminary evidence that higher levels of IL-6 at baseline are associated with a better outcome of VDZ therapy. This is in line with the trend showed in our previous study conducted in UC patients, aimed at evaluating the correlation between serum cytokines and mucosal healing [16]. On the other hand, a study by Soendergaard et al. [15] showed a correlation between lower levels of IL-6 at baseline and clinical response to VDZ after the induction. This difference could be related to the different endpoint: clinical response after the induction is certainly different in comparison with biochemical remission in terms of faecal calprotectin, which could be a surrogate marker of mucosal healing. Moreover, the study by Soendergaard et al. [15] included only IBD patients with a previous exposure to anti-TNF drugs: several studies demonstrated how VDZ is less effective in this population [24,26], and, furthermore, anti-TNF drugs are known to reduce IL-6 levels [14,54]. IL-6 is produced by innate immune and supporting stromal cells and activates adaptive T and B effector cells [51]. Following the findings by Zeissig et al. [46] described above, higher levels of IL-6 could be related to high activity of innate immunity in patients treated with VDZ. In this perspective, it is reasonable that a drug that exerts its function trough a modulation of innate immunity is more effective in patients with a high activation of the molecules involved in this process. However, it is worthy to note that additional evidence from other studies with a greater number of patients is needed to reach a reliable conclusion on the role of IL-6 in VDZ-treated patients.

This main strength of this study is the strong statistical correlation, confirmed at multivariate analysis, between clinical response to VDZ in IBD patients and the levels of IL-8 at baseline and the trend of IL-6. Moreover, the correlation with normal CRP and faecal calprotectin < 250 mg/kg could reflect a biochemical remission as well as an endoscopic response. Another important finding of this study, never demonstrated before, is the difference in terms of reliability of IL-6 and IL-8 in comparison with CRP or faecal calprotectin as prospective biomarkers of clinical response to VDZ, which could suggest the assessment of these cytokines in clinical practice, if confirmed in other studies with larger cohorts of UC patients. The difference highlighted between CD and UC patients in the present study is also particularly interesting and paves the way for future studies aimed at corroborating this preliminary evidence. Lastly, it is worthy to mention that we have included consecutive patients eligible to treatment with VDZ, reflecting a real-life practice.

The present study has some limitations. Firstly, the number of CD patients is relatively low; in this perspective, the results should be intended as exploratory in this specific cohort. Moreover, the assessment of VDZ trough levels could be interesting in order to evaluate the correlation between serum cytokines and VDZ pharmacokinetics, although this evaluation is not widely performed in real-life practice. Lastly, an endoscopic assessment at twelve months would improve the significance of the results, although faecal calprotectin < 250 mg/kg is currently well recognized as a marker of endoscopic remission [28,30]. However, performing colonoscopies would allow to collect biopsies, in order to evaluate even histological healing and tissue cytokine levels, which could be another important weakness of our study. On the other hand, we wish to note that the primary aim of this study, which was conceived as an exploratory search, was to identify a reliable and easy-to-perform biomarker capable to predict the clinical response to VDZ as early as possible. For this reason, we decided to focus our attention only on serum biomarkers, as blood is clearly far more accessible and less invasive than endoscopic evaluation with the collection of tissue bioptic specimens.

## 5. Conclusions

In conclusion, in patients with UC treated with VDZ, the assessment of serum levels of IL-6 and IL-8 values at baseline and after10 weeks of treatment may allow the prediction of clinical response at 12 months of therapy and thus may help clinicians to tailor personalized treatment strategies. Further studies are needed to validate these results on larger groups of patients with IBD undergoing treatment with VDZ.

## Figures and Tables

**Table 1:** Epidemiological features, clinical activity according to Harvey-Bradshaw index (HBI) and partial MAYO (pMAYO) score, biochemical activity and medications at baseline of the study population.

Characteristics	IBD	CD	UC
Number of patients	54	14	40
Age (years), median (range)	48 (18–80)	46 (18–80)	56 (20–76)
Sex (M/F)	14/40	10/4	10/30
Smoke (current/never/ex)	8/25/21	3/8/3	5/17/18
Years of illness, median (range)	14 (2–33)	18 (3–33)	11 (2–27)
Montreal classification	-	-	-
(CD: L1/L2/L3/L4; UC: E1/E2/E3)		1/1/12/1	3/16/21
Clinical activity (mean, 95% CI) (CD: HBI; UC: pMAYO)		7.1 (5.2–9)	5.3 (4.6–5.9)
Biochemical activity	-	-	-
Faecal calprotectin (mg/kg),	559 (382–816)	1620 (519–5064)	463 (314–683)
(geometric mean, 95% CI)	-	-	-
CRP (mg/L),	9.3 (6.9–12.8)	8.4 (1.5–46.39	9.8 (6.9–13.7)
(geometric mean, 95% CI)	-	-	-
ESR (positive/negative)	33/21	6/8	27/13
Concomitant medications	-	-	-
Mesalazine (yes/no, %)	48/6 (88.9%)	9/5 (64.3%)	39/1 (97.5%)
Systemic corticosteroids (yes/no, %)	33/21 (61.1%)	9/5 (64.3%)	24/16 (60.0%)
Azathioprine (yes/no, %)	8/46 (14.8%)	2/12 (14.3%)	6/34 (15.0%)

Abbreviations: male (M), female (F), inflammatory bowel disease (IBD); Crohn’s disease (CD); ulcerative colitis (UC); ileum (L1); colon (L2); ileum + colon (L3); upper gastrointestinal (L4); rectum (E1); left side (E2); extensive (E3); confidence interval (CI); C-reactive protein (CRP); erythrocyte sedimentation rate (ESR); higher than the upper limit of the reference (positive).

**Table 2:** IL-6 and IL-8 baseline values.

Parameters	Patients with CD (*n* = 14)	Patients with UC (*n* = 40)	*p-*Value
IL-6 (pg/mL), (geometric mean, range)	2.1 (0.6–7.1)	2.5 (0.2–29.8)	0.770
IL-8 (pg/mL), (geometric mean, range)	3.4 (OOR–29.7)	6.7 (OOR–61.4)	0.380

Abbreviations: interleukin (IL), Crohn’s disease (CD), ulcerative colitis (UC), out of range (OOR).

**Table 3:** Trend of the parameters at 3 and 12 months in the overall cohort and according to type of disease (CD and UC).

Parameters	T0	T1	*p-*Value (T1 vs. T0)	T2	*p-*Value (T2 vs. T0)
**Total cohort (*n* = 54)**					
Calprotectin (mg/kg), (geometric mean, 95% CI)	559 (382–816)	205 (129–325)	< 0.001	151 (83–275)	< 0.001
CRP (mg/L), (geometric mean, 95% CI)	9.3 (6.9–12.8)	5.1 (3.4–7.6)	0.007	4.4 (2.9–6.8)	0.006
ESR (positive/negative)	33/21	29/25	0.050	26/28	0.020
IL-6 (pg/mL), (median, 95% CI)	2.5 (1.5–3.5)	1.4 (1.1–1.9)	0.007	N/P	N/A
IL-8 (pg/mL), (median, 95% CI)	8.2 (5.2–12.2)	8.0 (5.1–9.6)	0.060	N/P	N/A
**CD (*n* = 14)**					
Calprotectin (mg/kg), (geometric mean, 95% CI)	1620 (519–5064)	608 (173–2129)	0.039	414 (45–3799)	0.017
CRP (mg/L), (geometric mean, 95% CI)	8.4 (1.5–46.3)	6.4 (2.0–20.3)	0.619	4.4 (1.0–18.8)	0.039
ESR (positive/negative)	6/8	5/9	1.000	4/10	0.500
IL-6 (pg/mL), (median, 95% CI)	1.6 (0.9–6.0)	2.0 (0.2–16.5)	0.414	N/P	N/A
IL-8 (pg/mL), (median, 95% CI)	5.8 (1.9–14.6)	8.9 (3.5–14.4)	0.970	N/P	N/A
**UC (*n* = 40)**					
FC (mg/kg), (geometric mean, 95% CI)	463 (314–683)	169 (103–277)	0.001	134 (70–255)	<0.001
CRP (mg/L), (geometric mean, 95% CI)	9.8 (6.9–13.7)	4.8 (3.1–7.5)	0.007	4.4 (2.8–7.0)	0.013
ESR (positive/negative)	27/13	24/16	0.250	22/18	0.063
IL-6 (pg/mL), (median, 95% CI)	2.5 (1.4–3.4)	1.4 (1.2–1.9)	0.009	N/P	N/A
IL-8 (pg/mL), (median, 95% CI)	8.3 (6.2–14.0)	8.0 (5.0–9.6)	0.032	N/P	N/A

Abbreviations: Before therapy (T0); after 10 weeks (T1); after twelve months (T2); Crohn’s disease (CD); C-reactive protein (CRP); erythrocyte sedimentation rate (ESR); interleukin (IL); not performed (N/P); not applicable (N/A); ulcerative colitis (UC).

**Table 4:** Adjusted OR of variables included in the multivariate regression analysis for the prediction of clinical response at T2 in patients with UC.

Variables	OR, 95% CI	*p*-Value
Baseline IL-8 > 8.6 pg/mL	14.74, 1.78–122.14	0.013
IL-6 reduction > 0.4 pg/mL	10.81, 1.58–73.68	0.015
Disease activity	0.70, 0.21–2.28	0.550
Disease extent (E3)	0.18, 0.02–1.35	0.097

Abbreviations: interleukin (IL), odds ratio (OR).
